# 2-(Benzotriazol-1-ylmethyl­amino)­benzoic acid

**DOI:** 10.1107/S160053680800740X

**Published:** 2008-03-20

**Authors:** Yao Wang, Mai-Hua Yin, Guo-Fang Zhang

**Affiliations:** a Key Laboratory of Applied Surface and Colloid Chemistry (Shaanxi Normal University), Ministry of Education, Xi’an 710062, People’s Republic of China

## Abstract

The title compound, C_14_H_12_N_4_O_2_, a new *N*,*O*,*N*′-tridentate ligand, is V-shaped with the mean plane through the benzotriazole system [planar to within 0.013 (2) Å] inclined by 67.7 (1)° to the mean plane through the benzene ring. In the mol­ecule there is an intra­molecular N—H⋯O hydrogen bond involving the amine H atom and the carbonyl O atom. In the crystal structure, symmtry-related mol­ecules are connected by inter­molecular O—H⋯N and C—H⋯O hydrogen bonds and C—H⋯π inter­actions.

## Related literature

For related literature, see: Trofimenko (1993 ▶); Zhang, Dou *et al.* (2007 ▶); Zhang *et al.* (2006 ▶); Zhang, Zhou *et al.* (2007 ▶).
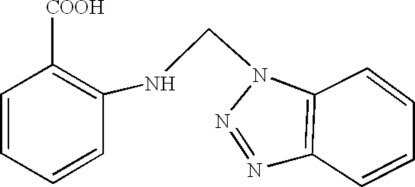


## Experimental

### 

#### Crystal data

C_14_H_12_N_4_O_2_
*M* *_r_* = 268.28
Monoclinic, 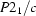

*a* = 10.225 (6) Å
*b* = 15.669 (8) Å
*c* = 8.098 (4) Å
β = 97.671 (7)°
*V* = 1285.8 (12) Å^3^
*Z* = 4
Mo *K*α radiation
μ = 0.10 mm^−1^
*T* = 291 (2) K
0.19 × 0.16 × 0.07 mm

#### Data collection

Bruker SMART CCD area-detector diffractometer
Absorption correction: multi-scan (*SADABS*; Sheldrick, 1996 ▶) *T*_min_ = 0.982, *T*_max_ = 0.993
9692 measured reflections
2388 independent reflections
1438 reflections with *I* > 2σ(*I*)
*R*_int_ = 0.053

#### Refinement

*R*[*F* ^2^ > 2σ(*F* ^2^)] = 0.047
*wR*(*F* ^2^) = 0.117
*S* = 1.02
2388 reflections
186 parameters
H atoms treated by a mixture of independent and constrained refinement
Δρ_max_ = 0.14 e Å^−3^
Δρ_min_ = −0.17 e Å^−3^

### 

Data collection: *SMART* (Bruker, 2007 ▶); cell refinement: *SAINT* (Bruker, 2007 ▶); data reduction: *SAINT*; program(s) used to solve structure: *SHELXS97* (Sheldrick, 2008 ▶); program(s) used to refine structure: *SHELXL97* (Sheldrick, 2008 ▶); molecular graphics: *SHELXTL* (Sheldrick, 2008 ▶); software used to prepare material for publication: *SHELXTL*.

## Supplementary Material

Crystal structure: contains datablocks I, global. DOI: 10.1107/S160053680800740X/su2045sup1.cif

Structure factors: contains datablocks I. DOI: 10.1107/S160053680800740X/su2045Isup2.hkl

Additional supplementary materials:  crystallographic information; 3D view; checkCIF report

## Figures and Tables

**Table 1:** Hydrogen-bond geometry (Å, °)

*D*—H⋯*A*	*D*—H	H⋯*A*	*D*⋯*A*	*D*—H⋯*A*
N4—H4*D*⋯O1	0.86 (2)	1.99 (3)	2.691 (3)	138 (2)
O2—H2⋯N3^i^	0.82	1.95	2.746 (3)	165
C7—H7*A*⋯O1^ii^	0.97	2.40	3.214 (3)	141
C12—H12⋯*Cg*2^iii^	0.93	2.94	3.836 (3)	163

Symmetry codes: (i) 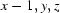; (ii) 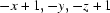; (iii) 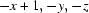. *Cg*2 is the centroid of the C1–C6 ring.

**Fig. 1.**
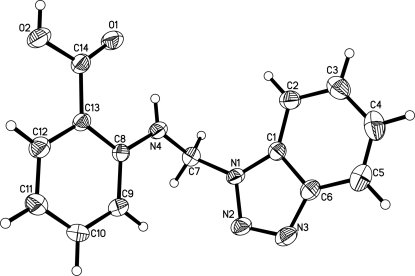
Molecular structure of the title compound showing the atom numbering scheme and dislacement ellipsoids drawn at the 50% probability level.

## supplementary crystallographic information

### Comment

In last decades, extensive investigations have been undertaken to design and synthesize pyrazole-based tridentate ligands, with the aim of mimiking structures and functions of some metalloenzymes (Trofimenko, 1993). Our interests have been focused on the design and syntheses of flexible N,*O*,*N* ligands derived from pyrazoles and triazoles since a certain flexibility might afford coordination versitality of the ligands. We have therefore designed and synthesized a number of such ligands as well as their transition-metal complexes (Zhang, Dou *et al.*, 2007; Zhang, Yin *et al.*, 2006; Zhang, Zhou *et al.*, 2007). Here we report on the structure of a new N,*O*,*N* tridentate ligand, 2-(benzotriazolylmethylamino)benzoic acid.

The molecular structure of the title compound is illustrated in Fig. 1. Details of the hydrogen bonding and C—H···\p interactions are given in Table 1. The molecule is V-shaped with the best plane through the benzotriazole moiety (planar to within 0.013 (2) Å) inclined by 67.7 (1)° to the best plane through the benzene ring (C8—C13). In the molecule there is an intra-molecular N—H···O hydrogen bond involving the amine (N4) hydrogen, H4D, and the carbonyl O-atom, O1 (Table 1).

In the crystal structure symmetry related molecules form dimers *via* C—H···π interactions involving C12—H12 and the benzene ring [(C1—C6 = *Cg*2^iii^]. Adjacent molecules are linked by O2—H2···N3^i^ hydrogen bonds to form zigzag chains parallel to the *a* axis, and these chains are further linked by C7—H7A···O1^ii^ intermolecular hydrogen bonds (Table 1).

### Experimental

The NH hydrogen atom was located from a difference Fourier map and freely refined, N—H = 0.86 (2) Å. The remainder of the H-atoms were included in calculated positions and treated as riding atoms: O—H = 0.82 Å and C—H = 0.93 - 0.97 \%A, with *U*_iso_(H) = 1.5*U*_eq_(O) and 1.2*U*_eq_(C).

### Crystal data

**Table:**

C_14_H_12_N_4_O_2_	*F*_000_ = 560
*M**_r_* = 268.28	*D*_x_ = 1.386 Mg m^−^^3^
Monoclinic, *P*2_1_/*c*	Mo *K*α radiation λ = 0.71073 Å
*a* = 10.225 (6) Å	Cell parameters from 1098 reflections
*b* = 15.669 (8) Å	θ = 2.4–21.9º
*c* = 8.098 (4) Å	µ = 0.10 mm^−^^1^
β = 97.671 (7)º	*T* = 291 (2) K
*V* = 1285.8 (12) Å^3^	Block, colourless
*Z* = 4	0.19 × 0.16 × 0.07 mm

### Data collection

**Table:**

Bruker SMART CCD area-detector diffractometer	2388 independent reflections
Radiation source: fine-focus sealed tube	1438 reflections with *I* > 2σ(*I*)
Monochromator: graphite	*R*_int_ = 0.053
*T* = 291(2) K	θ_max_ = 25.5º
φ and ω scans	θ_min_ = 2.4º
Absorption correction: multi-scan(SADABS; Sheldrick, 1996)	*h* = −12→12
*T*_min_ = 0.982, *T*_max_ = 0.993	*k* = −18→18
9692 measured reflections	*l* = −9→9

### Refinement

**Table:**

Refinement on *F*^2^	Secondary atom site location: difference Fourier map
Least-squares matrix: full	Hydrogen site location: inferred from neighbouring sites
*R*[*F*^2^ > 2σ(*F*^2^)] = 0.047	H atoms treated by a mixture of independent and constrained refinement
*wR*(*F*^2^) = 0.117	*w* = 1/[σ^2^(*F*_o_^2^) + (0.0885*P*)^2^ + 0.2074*P*] where *P* = (*F*_o_^2^ + 2*F*_c_^2^)/3
*S* = 1.02	(Δ/σ)_max_ < 0.001
2388 reflections	Δρ_max_ = 0.14 e Å^−^^3^
186 parameters	Δρ_min_ = −0.17 e Å^−^^3^
Primary atom site location: structure-invariant direct methods	Extinction correction: none

### Special details

**Table:**

Geometry. All e.s.d.'s (except the e.s.d. in the dihedral angle between two l.s. planes)are estimated using the full covariance matrix. The cell e.s.d.'s are takeninto account individually in the estimation of e.s.d.'s in distances, anglesand torsion angles; correlations between e.s.d.'s in cell parameters are onlyused when they are defined by crystal symmetry. An approximate (isotropic)treatment of cell e.s.d.'s is used for estimating e.s.d.'s involving l.s. planes.
Refinement. Refinement of *F*^2^ against ALL reflections. The weighted *R*-factor *wR* andgoodness of fit *S* are based on *F*^2^, conventional *R*-factors *R* are basedon *F*, with *F* set to zero for negative *F*^2^. The threshold expression of*F*^2^ > σ(*F*^2^) is used only for calculating *R*-factors(gt) *etc*. and isnot relevant to the choice of reflections for refinement. *R*-factors basedon *F*^2^ are statistically about twice as large as those based on *F*, and *R*-factors based on ALL data will be even larger.

### Fractional atomic coordinates and isotropic or equivalent isotropic displacement parameters (Å^2^)

**Table:**

	*x*	*y*	*z*	*U*_iso_*/*U*_eq_	
O1	0.37616 (15)	0.01777 (11)	0.3049 (2)	0.0594 (5)	
O2	0.23657 (15)	0.05486 (12)	0.0817 (2)	0.0609 (5)	
H2	0.1845	0.0294	0.1327	0.091*	
N1	0.85744 (16)	0.01883 (12)	0.3449 (2)	0.0414 (5)	
N2	0.95246 (18)	0.05227 (13)	0.2642 (2)	0.0511 (5)	
N3	1.02834 (18)	−0.00961 (14)	0.2240 (2)	0.0539 (6)	
N4	0.62976 (19)	0.06560 (14)	0.3142 (3)	0.0488 (6)	
C1	0.8708 (2)	−0.06775 (14)	0.3566 (3)	0.0393 (5)	
C2	0.8006 (2)	−0.13078 (16)	0.4280 (3)	0.0506 (6)	
H2A	0.7279	−0.1185	0.4818	0.061*	
C3	0.8460 (3)	−0.21219 (17)	0.4134 (3)	0.0635 (8)	
H3	0.8022	−0.2567	0.4586	0.076*	
C4	0.9558 (3)	−0.23133 (19)	0.3332 (4)	0.0723 (9)	
H4	0.9827	−0.2878	0.3270	0.087*	
C5	1.0235 (3)	−0.16907 (19)	0.2644 (4)	0.0657 (8)	
H5	1.0957	−0.1820	0.2103	0.079*	
C6	0.9808 (2)	−0.08513 (16)	0.2776 (3)	0.0462 (6)	
C7	0.7583 (2)	0.07434 (15)	0.4056 (3)	0.0479 (6)	
H7A	0.7538	0.0612	0.5218	0.057*	
H7B	0.7864	0.1333	0.3995	0.057*	
C8	0.5883 (2)	0.10492 (13)	0.1642 (3)	0.0377 (5)	
C9	0.6766 (2)	0.15121 (14)	0.0805 (3)	0.0488 (6)	
H9	0.7650	0.1547	0.1257	0.059*	
C10	0.6343 (2)	0.19133 (15)	−0.0668 (3)	0.0578 (7)	
H10	0.6946	0.2223	−0.1192	0.069*	
C11	0.5051 (3)	0.18711 (16)	−0.1397 (3)	0.0595 (7)	
H11	0.4774	0.2150	−0.2396	0.071*	
C12	0.4182 (2)	0.14051 (15)	−0.0609 (3)	0.0489 (6)	
H12	0.3310	0.1361	−0.1103	0.059*	
C13	0.4559 (2)	0.09968 (13)	0.0902 (3)	0.0372 (5)	
C14	0.3554 (2)	0.05360 (15)	0.1695 (3)	0.0441 (6)	
H4D	0.568 (2)	0.0420 (16)	0.360 (3)	0.068 (9)*	

### Atomic displacement parameters (Å^2^)

**Table:**

	*U*^11^	*U*^22^	*U*^33^	*U*^12^	*U*^13^	*U*^23^
O1	0.0421 (10)	0.0791 (13)	0.0586 (12)	−0.0032 (9)	0.0122 (9)	0.0203 (10)
O2	0.0344 (9)	0.0840 (14)	0.0641 (12)	−0.0085 (9)	0.0054 (9)	0.0151 (10)
N1	0.0287 (10)	0.0482 (12)	0.0475 (12)	−0.0040 (9)	0.0062 (9)	0.0045 (9)
N2	0.0332 (11)	0.0629 (14)	0.0563 (13)	−0.0067 (10)	0.0030 (10)	0.0090 (11)
N3	0.0342 (11)	0.0726 (15)	0.0552 (13)	−0.0018 (11)	0.0078 (10)	0.0039 (11)
N4	0.0306 (11)	0.0626 (14)	0.0532 (14)	−0.0003 (10)	0.0050 (10)	0.0160 (11)
C1	0.0350 (12)	0.0427 (14)	0.0385 (13)	−0.0007 (11)	−0.0010 (10)	−0.0001 (11)
C2	0.0447 (14)	0.0559 (16)	0.0507 (16)	−0.0061 (13)	0.0050 (12)	0.0067 (13)
C3	0.0670 (19)	0.0527 (18)	0.0670 (19)	−0.0078 (15)	−0.0056 (15)	0.0064 (14)
C4	0.077 (2)	0.0529 (18)	0.082 (2)	0.0130 (16)	−0.0071 (18)	−0.0084 (16)
C5	0.0532 (17)	0.073 (2)	0.070 (2)	0.0111 (15)	0.0053 (14)	−0.0125 (16)
C6	0.0351 (13)	0.0555 (16)	0.0465 (15)	−0.0006 (12)	0.0000 (11)	−0.0029 (12)
C7	0.0418 (14)	0.0496 (15)	0.0520 (15)	0.0025 (12)	0.0055 (11)	0.0017 (12)
C8	0.0368 (12)	0.0336 (12)	0.0436 (14)	0.0024 (10)	0.0094 (11)	0.0011 (10)
C9	0.0360 (13)	0.0501 (15)	0.0611 (17)	−0.0038 (11)	0.0100 (12)	0.0061 (13)
C10	0.0503 (16)	0.0570 (17)	0.0695 (19)	−0.0034 (13)	0.0205 (14)	0.0208 (14)
C11	0.0541 (17)	0.0657 (18)	0.0584 (17)	−0.0023 (14)	0.0061 (13)	0.0207 (14)
C12	0.0406 (13)	0.0565 (16)	0.0488 (15)	−0.0017 (12)	0.0030 (12)	0.0026 (13)
C13	0.0320 (12)	0.0379 (13)	0.0429 (14)	0.0023 (10)	0.0096 (10)	−0.0019 (10)
C14	0.0360 (13)	0.0460 (15)	0.0512 (16)	0.0025 (11)	0.0088 (12)	−0.0011 (12)

### Geometric parameters (Å, °)

**Table:**

O1—C14	1.225 (3)	C4—C5	1.358 (4)
O2—C14	1.323 (3)	C4—H4	0.9300
O2—H2	0.8200	C5—C6	1.394 (4)
N1—N2	1.347 (2)	C5—H5	0.9300
N1—C1	1.365 (3)	C7—H7A	0.9700
N1—C7	1.470 (3)	C7—H7B	0.9700
N2—N3	1.310 (3)	C8—C9	1.402 (3)
N3—C6	1.371 (3)	C8—C13	1.408 (3)
N4—C8	1.377 (3)	C9—C10	1.367 (3)
N4—C7	1.426 (3)	C9—H9	0.9300
N4—H4D	0.86 (2)	C10—C11	1.375 (3)
C1—C2	1.391 (3)	C10—H10	0.9300
C1—C6	1.393 (3)	C11—C12	1.372 (3)
C2—C3	1.368 (3)	C11—H11	0.9300
C2—H2A	0.9300	C12—C13	1.389 (3)
C3—C4	1.402 (4)	C12—H12	0.9300
C3—H3	0.9300	C13—C14	1.472 (3)
			
C14—O2—H2	109.5	N4—C7—N1	113.5 (2)
N2—N1—C1	110.39 (18)	N4—C7—H7A	108.9
N2—N1—C7	120.44 (19)	N1—C7—H7A	108.9
C1—N1—C7	129.17 (18)	N4—C7—H7B	108.9
N3—N2—N1	108.78 (18)	N1—C7—H7B	108.9
N2—N3—C6	108.27 (18)	H7A—C7—H7B	107.7
C8—N4—C7	124.7 (2)	N4—C8—C9	121.1 (2)
C8—N4—H4D	114.7 (18)	N4—C8—C13	121.0 (2)
C7—N4—H4D	119.7 (18)	C9—C8—C13	118.0 (2)
N1—C1—C2	133.0 (2)	C10—C9—C8	120.7 (2)
N1—C1—C6	104.0 (2)	C10—C9—H9	119.6
C2—C1—C6	123.0 (2)	C8—C9—H9	119.6
C3—C2—C1	115.2 (2)	C9—C10—C11	121.8 (2)
C3—C2—H2A	122.4	C9—C10—H10	119.1
C1—C2—H2A	122.4	C11—C10—H10	119.1
C2—C3—C4	122.8 (3)	C12—C11—C10	118.1 (2)
C2—C3—H3	118.6	C12—C11—H11	121.0
C4—C3—H3	118.6	C10—C11—H11	121.0
C5—C4—C3	121.3 (3)	C11—C12—C13	122.3 (2)
C5—C4—H4	119.3	C11—C12—H12	118.9
C3—C4—H4	119.3	C13—C12—H12	118.9
C4—C5—C6	117.7 (3)	C12—C13—C8	119.1 (2)
C4—C5—H5	121.2	C12—C13—C14	118.8 (2)
C6—C5—H5	121.2	C8—C13—C14	122.1 (2)
N3—C6—C1	108.6 (2)	O1—C14—O2	121.7 (2)
N3—C6—C5	131.4 (2)	O1—C14—C13	124.6 (2)
C1—C6—C5	120.0 (2)	O2—C14—C13	113.8 (2)
			
C1—N1—N2—N3	−0.6 (2)	C8—N4—C7—N1	−81.5 (3)
C7—N1—N2—N3	179.61 (18)	N2—N1—C7—N4	109.0 (2)
N1—N2—N3—C6	0.8 (2)	C1—N1—C7—N4	−70.8 (3)
N2—N1—C1—C2	179.3 (2)	C7—N4—C8—C9	6.5 (3)
C7—N1—C1—C2	−0.9 (4)	C7—N4—C8—C13	−173.6 (2)
N2—N1—C1—C6	0.1 (2)	N4—C8—C9—C10	−178.8 (2)
C7—N1—C1—C6	179.9 (2)	C13—C8—C9—C10	1.3 (3)
N1—C1—C2—C3	−179.9 (2)	C8—C9—C10—C11	−0.9 (4)
C6—C1—C2—C3	−0.9 (3)	C9—C10—C11—C12	−0.5 (4)
C1—C2—C3—C4	0.3 (4)	C10—C11—C12—C13	1.5 (4)
C2—C3—C4—C5	−0.2 (4)	C11—C12—C13—C8	−1.1 (3)
C3—C4—C5—C6	0.6 (4)	C11—C12—C13—C14	177.8 (2)
N2—N3—C6—C1	−0.8 (2)	N4—C8—C13—C12	179.8 (2)
N2—N3—C6—C5	179.0 (2)	C9—C8—C13—C12	−0.3 (3)
N1—C1—C6—N3	0.4 (2)	N4—C8—C13—C14	1.0 (3)
C2—C1—C6—N3	−178.9 (2)	C9—C8—C13—C14	−179.1 (2)
N1—C1—C6—C5	−179.4 (2)	C12—C13—C14—O1	−177.4 (2)
C2—C1—C6—C5	1.3 (3)	C8—C13—C14—O1	1.4 (3)
C4—C5—C6—N3	179.1 (2)	C12—C13—C14—O2	1.6 (3)
C4—C5—C6—C1	−1.2 (4)	C8—C13—C14—O2	−179.6 (2)

### Hydrogen-bond geometry (Å, °)

**Table:**

*D*—H···*A*	*D*—H	H···*A*	*D*···*A*	*D*—H···*A*
N4—H4D···O1	0.86 (2)	1.99 (3)	2.691 (3)	138 (2)
O2—H2···N3^i^	0.82	1.95	2.746 (3)	165
C7—H7A···O1^ii^	0.97	2.40	3.214 (3)	141
C12—H12···Cg2^iii^	0.93	2.94	3.836 (3)	163

Symmetry codes:  (i) *x*−1, *y*, *z*; (ii) −*x*+1, −*y*, −*z*+1; (iii) −*x*+1, −*y*, −*z*.
